# Association of retinal fractal dimension and vessel tortuosity with impaired renal function among healthy Chinese adults

**DOI:** 10.3389/fmed.2022.925756

**Published:** 2022-09-02

**Authors:** Linbin Wu, Xia Gong, Wei Wang, Lei Zhang, Jiachen Zhou, Xi Ming, Meng Yuan, Wenyong Huang, Lanhua Wang

**Affiliations:** ^1^The First People’s Hospital of Zhaoqing, Zhaoqing, China; ^2^Zhongshan Ophthalmic Center, Guangdong Provincial Clinical Research Center for Ocular Diseases, State Key Laboratory of Ophthalmology, Guangdong Provincial Key Laboratory of Ophthalmology and Visual Science, Sun Yat-sen University, Guangzhou, China

**Keywords:** OCTA, renal function, eGFR, fractal dimension, blood vessel tortuosity

## Abstract

**Purpose:**

This study investigated the association of retinal fractal dimension (FD) and blood vessel tortuosity (BVT) with renal function [assessed by estimated glomerular filtrate rate (eGFR)] in healthy Chinese adults using swept-source optical coherence tomographic angiography (SS-OCTA).

**Materials and methods:**

This cross-sectional study was conducted among ocular treatment–naïve healthy participants from Guangzhou, China. FD and BVT in the superficial capillary plexus and deep capillary plexus were measured by SS-OCTA with a 3 × 3 macula model. eGFR was calculated using the Xiangya equation, and impaired renal function (IRF) was defined as eGFR = 90 mL/min/1.73 m^2^. Linear regression was performed to evaluate the relationships between SS-OCTA metrics and renal function.

**Results:**

A total of 729 participants with a mean age of 57.6 ± 9.1 years were included in the final analysis. Compared to participants with normal renal function, those with IRF had lower FD both in the superficial capillary plexus (1.658 ± 0.029 vs. 1.666 ± 0.024, *p* = 0.001) and deep capillary plexus (1.741 ± 0.016 vs. 1.746 ± 0.016, *p* = 0.0003), while the deep BVT was larger in participants with IRF than those with normal renal function (1.007 ± 0.002 vs. 1.006 ± 0.002, *p* = 0.028). The superficial FD was linearly and positively associated with eGFR after adjusting for confounders (β = 0.2257; 95% CI 0.0829–0.3685; *p* = 0.002), while BVT was not associated with eGFR (all *p* ≥ 0.05).

**Conclusion:**

The patients with IRF had lower FD and larger BVT than those with normal renal function. The superficial FD decreased linearly with renal function deterioration. Our study suggests that the retinal microvasculature can represent a useful indicator of subclinical renal microvascular abnormalities and serve as a useful non-invasive assessment to predict and monitor the progression of renal function.

## Introduction

Chronic kidney disease (CKD) is a major public health hazard that is strongly associated with diabetes, hypertension and, eventually, death (1). The number of people with CKD is estimated to rise sharply as populations (2) age, and CKD may become the fifth most common cause of death worldwide by 2040 (3) if timely control and treatment strategies are not provided. Thus, it is important to identify patients with CKD at an early stage or those at high risk of developing CKD (4) to reduce unnecessary treatment and costs. However, early diagnosis of CKD has long been medically challenging, as most patients with CKD are diagnosed after renal function has been significantly lost. In addition, the predictive performance of common biomarkers for CKD development and progression, including serum creatinine (Scr), cystatin C and proteinuria, is limited (5). Therefore, identifying new biomarkers of renal function can be helpful in the early detection of CKD and the implementation of timely interventions (6).

Microvascular damage plays a key role in the progression and development of CKD (7). Considering that the retina and kidney share similar anatomic, physiological and pathogenic vascular tissue characteristics and retinal vessels are the only visualizable vascular tissues that can be non-invasively observed *in vivo* (8), it is possible to predict and monitor changes and development of renal function *via* retinal vessels. Several studies have indicated that metrics of the retinal macrovasculature (including central retinal arteriolar equivalent and central retinal venular equivalent), measured by fundus photography, are closely associated with renal function (9–11). However, the retinal microvasculature, which is more representative of retinal microcirculation, cannot be assessed *via* fundus photography (8).

The advent of optical coherence tomographic angiography (OCTA) has provided an opportunity to non-invasively and quantitatively evaluate the retinal microvasculature. By using OCTA, several studies have evaluated the relationship between renal function and retinal vessel density (VD) (12). Besides retinal VD, a new class of geometric parameters which are potentially more indicative of the quality of the retinal microvasculature, including fractal dimension (FD) and blood vessel tortuosity (BVT) (13–16), have been developed by a range of computer-based programs. However, to our knowledge, no study has yet investigated the association of renal function with FD and BVT.

The association of renal function with retinal microvascular parameters is not fully understood. However, analyses of GFR-VD association have proven that there is a positive GFR-VD association among patients with diabetes (17). This current study aimed to evaluate the association of renal function with retinal FD and BVT in a large sample of Chinese adults.

## Materials and methods

### Participants

This cross-sectional study was conducted at Zhongshan Ophthalmic Center, which is affiliated with Sun Yat-sen University, Guangzhou. Ocular treatment–naïve adults aged 30–80 years from Guangzhou communities were recruited for this study between 9 July 2018 and 1 January 2019. Patients with a medical history of ocular diseases, best-corrected visual acuity worse than Snellen 20/200, spherical equivalent > –12D, astigmatism > 4D, OCTA images of poor quality and/or axial length (AL) >30 mm were excluded from this study. The Ethics Committee of Zhongshan Ophthalmic Center approved the study protocol (No: 2017KYPJ094). The study was conducted according to the tenets of the Helsinki Declaration, and a signed informed consent form was obtained from all the participants.

### Questionnaire and ocular examinations

Each participant completed detailed ocular examinations: uncorrected visual acuity and best-corrected visual acuity with the Early Treatment of Diabetic Retinopathy Study (ETDRS) LogMAR E chart (Precision Vision, Villa Park, IIIinois, United States) at a distance of four meters; non-contact intraocular pressure measurement (Topcon CT-80A, Topcon, Tokyo, Japan); anterior segment and fundus examination using a slit lamp (BQ-900, Haag-Streit, Switzerland); autorefraction (KR-8800, Topcon, Japan); ocular biometric measurements, including corneal curvature, anterior chamber depth, lens thickness and AL, with Lenstar LS900 (Haag-Streit AG, Koeniz, Switzerland); fundus photography; OCT; and OCTA. After the pupil was fully dilated with 0.5% topiramide and 0.5% phenylephrine eye drops, standard 7-field 45 color retinal photographs were taken using a digital fundus camera (CR-2; Canon, Tokyo, Japan) according to ETDRS standards.

### Swept-source optical coherence tomographic angiography imaging

All the participants underwent OCTA imaging carried out by the same experienced technicians with swept-source OCTA (SS-OCT; DRI OCT Triton, Topcon, Tokyo, Japan), which uses a central wavelength of 1,050 nm and a speed of 100,000 A-scans per second. The standardized 3 × 3 angiography model was used to obtain images of the macula area a half hour after pupil dilatation. Then the FD and BVT in the SCP and DCP in the macula area were automatically calculated by the built-in software (IMAGEnet 6). The image quality of each scan, which ranged from 0 to 100, was automatically graded by IMAGEnet 6 (18).

The magnification effect by axial length was adjusted using Littmann’s method and the Bennett formula (19). FD% was measured by using Fiji software to quantify retinal perfusion. Vessel images were binarized by the Phansalkar threshold method with a radius of 2–4 pixels (20). Then FD% in the reversed images was quantified by analyzing the particle module (21). The FD% in ETDRS grids was measured.

BVT was calculated using ImageJ software (National Institutes of Health, Bethesda, United States). An adjusted threshold tool was used to highlight the blood vessels and reduce the noise. After vessel binarization, BVT was defined as the ratio of shortest path to straight-line length (22, 23).

FD% and BVT in the whole image, the parafoveal region and five subregions (superior, inferior, nasal, temporal and central fields) were reported separately. The data were obtained and analyzed independently by two trained ophthalmologists who were not aware of patients’ information. The detailed method of image analysis and sample images have been described in our previous published articles (24, 25).

### Questionnaire and systemic evaluation

Each participant filled out a standardized questionnaire with the following sections: personal information, lifestyle risk factors, history of systemic and ocular disease, medication and history of surgeries. Systolic and diastolic blood pressure were measured by an automatic electronic sphygmomanometer (HBP-9020, Omron, Osaka, Japan). Height and weight were measured without shoes and heavy clothes, and body mass index was defined as weight (kg) divided by height square (m^2^). Scr, glycosylated hemoglobin, total cholesterol, high-density lipoprotein cholesterol, low-density lipoprotein cholesterol, triglyceride and microalbuminuria (MAU) were tested with standardized procedures. The eGFR was calculated based on the Xiangya equation (13), and a participant with an eGFR < 90 mL/min/1.73 m^2^ was considered to have impaired renal function (IRF).

### Statistical analysis

Data from the right eye were included in the statistical analysis. All statistical analyses were performed using Stata 10.0 (Stata Corp., College Station, TX). The normal distribution was determined by the Shapiro–Wilke test. Continuous variables were presented as mean ± standard deviation (SD), and Student’s *t*-test was used to determine statistically significant differences. Categorical data were presented as proportions, and Pearson X^2^ or Fisher’s exact test was used to determine statistically significant differences. A univariable linear regression model was used to evaluate the relationship between renal function and retinal microvascular parameters (FD and BVT). Multivariate models were further established by adjusting for known confounders of the retinal vasculature, including age, sex, current smoker, systolic blood pressure, diastolic blood pressure, cholesterol, glycosylated hemoglobin, triglyceride, high-density lipoprotein cholesterol and low-density lipoprotein cholesterol. Differences were considered statistically significant with a *p* < 0.05.

## Results

A total of 729 participants with an average age of 57.6 ± 9.1 years were included in the final analysis. The basic demographic and clinical characteristics are shown in Table 1. In general, 586 (80.4%) of the 729 participants had normal renal function, while 143 (19.6%) had IRF. The mean value of eGFR was 104.1 ± 15.6 mL/min/1.73 m^2^, and mean MAU was 1.0 ± 2.9 mg/ml. There were significant differences in sex, age, systolic blood pressure, Scr, eGFR and MAU between participants with and without IRF (all *p* < 0.05).

**TABLE 1:** Demographic and clinical characteristics of study participants.

Characteristics	Overall	Normal	Impaired	*P* *
No of patients	729	586 (80.4%)	143 (19.6%)	–
Female, %	550 (75.5%)	456 (77.8%)	94 (65.7%)	**0.003**
Age, year	57.6 (9.1)	56.5 (9.2)	62.3 (7.2)	**<0.001**
HbA1c, %	5.8 (0.6)	5.8 (0.6)	5.8 (0.5)	0.414
BMI, kg/m^2^	23.2 (3.0)	23.2 (3.1)	23.4 (3.0)	0.347
SBP, mmHg	125.2 (18.7)	124.2 (18.4)	129.6 (19.5)	**0.002**
DBP, mmHg	68.8 (10.7)	68.6 (10.6)	69.8 (10.7)	0.222
Total cholesterol, mmol/L	5.2 (1.0)	5.2 (1.0)	5.3 (1.0)	0.444
HDL-c, mmol/L	1.4 (0.4)	1.4 (0.4)	1.4 (0.4)	0.783
LDL-c, mmol/L	3.3 (0.8)	3.3 (0.8)	3.3 (0.9)	0.496
Triglycerides, mmol/L	2.1 (1.5)	2.2 (1.6)	2.0 (1.2)	0.154
Serum creatinine, μmol/L	68.5 (14.6)	64.1 (11.4)	86.4 (12.8)	**<0.001**
eGFR, ml/min/1.73 m^2^	104.1 (15.6)	109.9 (11.0)	80.5 (7.6)	**<0.001**
Microalbuminuria, mg/mL	1.0 (2.9)	0.9 (2.1)	1.5 (5.0)	**0.026**
Intraocular pressure, mmHg	15.7 (2.3)	15.8 (2.3)	15.4 (2.2)	0.122
CCT, μm	538.7 (30.8)	538.1 (30.7)	541.3 (31.4)	0.257
Axial length, mm	23.5 (0.9)	23.5 (1.0)	23.3 (0.8)	0.140

Data are expressed as the mean ± SD or %. Impaired renal function was defined as eGFR < 90 mL/min/1.73 m^2^. HbA1c, glycosylated hemoglobin; BMI, body mass index; SBP, systolic blood pressure; DBP, diastolic blood pressure; HDL-c, High-density lipoprotein cholesterol; LDL-c, Low-density lipoprotein cholesterol; eGFR, estimated glomerular filtration rate; CCT, central corneal thickness. *Bold indicates statistical significance.

**FIGURE 1:**
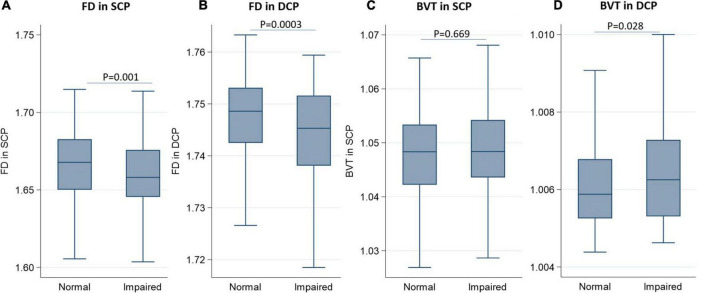
Boxplots showing the distribution of fractal dimension and blood vessel tortuosity in the superficial capillary plexus and deep capillary plexus stratified by renal function. **(A)** Fractal dimension in the superficial capillary plexus; **(B)** fractal dimensionin the deep capillary plexus; **(C)** blood vessel tortuosity in the superficial capillary plexus; **(D)** blood vessel tortuosity in the deep capillary plexus.

Table 2 and Figure 1 show the distribution of FD and BVT stratified by renal function status. The FD in the SCP decreased as renal function declined, with a mean value (SD) of 1.666 (0.024) for healthy participants, which decreased to 1.658 (0.029) for participants with IRF (*p* = 0.001). Correspondingly, the mean value (SD) of FD in the DCP also decreased from 1.746 (0.013) for healthy participants to 1.741 (0.016) for participants with IRF (*p* = 0.0003). The mean value (SD) of BVT in the DCP increased from 1.006 (0.002) for healthy participants to 1.007 (0.002) for participants with IRF (*p* = 0.028), while BVT in the SCP did not show a statistically significant difference between the groups (*p* = 0.669).

**TABLE 2:** Fractal dimension and tortuosity in participants stratified by estimated glomerular filtration rate.

	Overall	Normal	Impaired	*P* *
FD in SCP	1.665 (0.025)	1.666 (0.024)	1.658 (0.029)	**0.001**
FD in DCP	1.745 (0.014)	1.746 (0.013)	1.741 (0.016)	**0.003**
BVT in SCP	1.047 (0.009)	1.047 (0.009)	1.048 (0.010)	0.669
BVT in DCP	1.006 (0.002)	1.006 (0.002)	1.007 (0.002)	**0.028**

FD, fractal dimension; BVT, blood vessel tortuosity; SCP, superficial capillary plexus; DCP, deep capillary plexus. *Bold indicates statistical significance.

**FIGURE 2:**
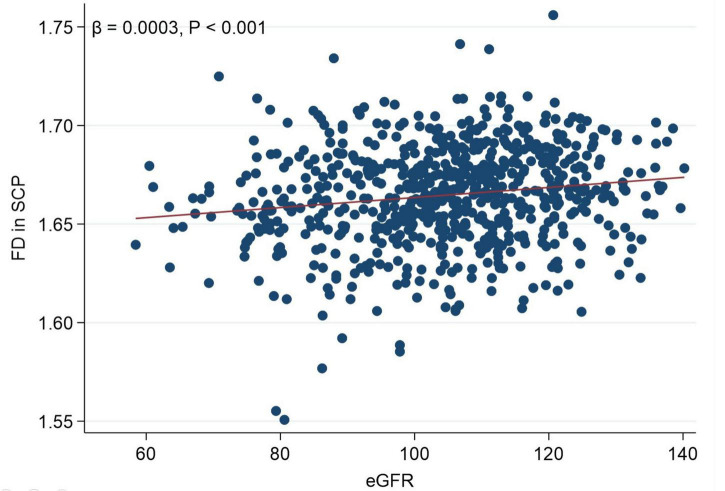
Scattergram showing linear correlation between fractal dimension in the superficial capillary plexus and estimated glomerular filtration rate.

Figure 2 presents a linear correlation between superficial FD and eGFR. Table 3 shows the results of the univariable linear regression of FD and BVT with eGFR. The FD in the SCP (β = 0.2555; 95% CI 0.1340–0.3770; *p* < 0.001) was positively associated with eGFR (Table 3 and Figure 2). Similarly, the FD in the DCP (β = 0.1295; 95% CI 0.0666–0.1924; *p* < 0.001) was also positively associated with eGFR. The BVT in the SCP and the DCP was not associated with eGFR.

**TABLE 3:** Univariable linear regression of renal function with fractal dimension and blood vessel tortuosity.

Per 1 eGFR increase	Coefficient (95% CI)			*P* *
	Estimate	Lower	Upper	
FD in SCP, × 10^–3^	0.2555	0.1340	0.3770	**<0.001**
FD in DCP, × 10^–3^	0.1295	0.0666	0.1924	**<0.001**
BVT in SCP, × 10^–3^	–0.0124	–0.0550	0.0302	0.567
BVT in DCP, × 10^–3^	–0.0076	–0.0168	0.0016	0.103

eGFR, estimated glomerular filtration rate; 95% CI, 95% confidential interval; FD, fractal dimension; BVT, blood vessel tortuosity; SCP, superficial capillary plexus; DCP, deep capillary plexus. *Adjusted for age, sex, current smoker, systolic blood pressure, diastolic blood pressure, cholesterol, glycosylated hemoglobin, tryglyceride, high-density lipoprotein cholesterol and low-density lipoprotein cholesterol. *Bold indicates statistical significance.

Table 4 shows the results of the multivariable linear regression of FD and BVT with eGFR, and the findings showed that FD in the SCP was still positively associated with eGFR (β = 0.2257; 95% CI 0.0829–0.3685; *p* = 0.002), while FD in the DCP, BVT in the SCP and DCP was not related to eGFR (all *p* > 0.05).

**TABLE 4:** Multivariable linear regression of renal function with measurements of fractal dimension and blood vessel tortuosity.

Per 1 eGFR increase	Coefficient (95% CI)			*P* *
	Estimate	Lower	Upper	
FD in SCP, × 10^–3^	0.2257	0.0829	0.3685	**0.002**
FD in DCP, × 10^–3^	0.0151	–0.0582	0.0884	0.686
BVT in SCP, × 10^–3^	–0.0029	–0.0539	0.0482	0.913
BVT in DCP, × 10^–3^	–0.0005	–0.0114	0.0103	0.922

eGFR, estimated glomerular filtration rate; 95%CI, 95% confidential interval; FD, fractal dimension; BVT, blood vessel tortuosity; SCP, superficial capillary plexus; DCP, deep capillary plexus. *Adjusted for age, sex, current smoker, systolic blood pressure, diastolic blood pressure, cholesterol, glycosylated hemoglobin, tryglyceride, high-density lipoprotein cholesterol and low-density lipoprotein cholesterol. *Bold indicates statistical significance.

## Discussion

The number of patients with CKD is increasing, which endangers public health and consumes tremendous medical resources. Advanced CKD is irreversible, so early detection and intervention of the occurrence and development of CKD is very important (1, 2). However, most of the traditional biomarkers for CKD need to be obtained through invasive blood tests or renal biopsy, and their value for early CKD diagnosis is limited (5). In this cross-sectional study, we first investigated the association of FD and BVT in two capillary plexuses (superficial and deep) with eGFR among participants with IRF and those with normal renal function. Our main results are summarized as follows: (1) participants with IRF had smaller FD in the SCP and DCP compared with healthy participants; (2) BVT in the DCP was larger for participants with IRF than participants with normal renal function; (3) there was a positive linear association between FD in the SCP and the eGFR, suggesting that retinal capillaries became non-complex as renal function deteriorated; and (4) BVT was not linearly associated with eGFR. This current study demonstrates that changes in the retinal microvasculature using OCTA may potentially be useful in identification and screening for those at high risk of CKD.

Retinal FD, which quantitatively describes the complexity of branching retinal vessels and the density of the whole retinal vascular system, is considered to be the early biomarker of vascular changes and is helpful in predicting the development and progression of ocular diseases (26, 27). There have been studies exploring the association between retinal FD and renal function based on digital fundus imaging or fundus fluorescein angiography. Some studies indicated a positive association of FD with CKD and GFR (11, 28, 29), while others showed no such association (30, 31). However, these traditional imaging methods are often invasive or cannot provide high resolution images of the retinal microvascular network at different layers of capillary plexus, so they cannot detect microvascular changes at the early stages (32).

The emergence of OCTA enables non-invasive assessment of renal microvascular changes. However, only a few studies have investigated the relationship between renal function and OCTA parameters, most of which focus on VD (6, 17) and foveal avascular zone. To our knowledge, only one study has reported no associations between FD and eGFR among 184 Chinese adults with diabetes (12). In the current study, we first assessed the relationship between renal function and FD in the SCP and DCP among healthy Chinese adults and found that participants with IRF had smaller superficial and deep FD than healthy participants, and the superficial FD linearly decreased with renal function deterioration. The results of the current study and previous studies of fundus images indicate that a loss of the retinal microvasculature complexity is closely associated with reduced renal function, and FD may serve as a predictive biomarker for renal function loss. In fact, retinal FD is extremely sensitive to other conditions involving vascular and neuronal health (33, 34). However, the cross-sectional design of the current study, with participants from communities in a single city, limited a deeper analysis of the retinal microvasculature and renal function; thus, further longitudinal studies with larger and diverse study samples are needed.

Increased BVT is considered to be related to the autoregulation of damaged blood vessels (35), and retinal BVT is considered to be the earliest identifiable vascular change in many retinal diseases and was associated with many systemic factors (35–39). The association of arterior and venular BVT and renal function based on fundus graphs is well established. However, there are no OCTA studies about BVT and renal function. In the current study, first, participants with IRF were shown to be more tortuous in the DCP compared to the healthy participants. Difference in organizational structural characteristics of the DCP and SCP may partly explain this phenomenon. The DCP is the termination of retinal capillary units and is more vulnerable to be affected (40). We speculate that the tortuosity of these capillaries may be due to the selective non-perfusion of DCP. However, we did not find a linear correlation between BVT and GFR, suggesting that a non-linear relationship may exist between BVT and GFR, which needs to be further studied in a larger study.

Many studies have attributed the significant association of microvascular renal pathology with microvascular retinopathy, two common microvascular diseases, to the shared anatomic, physiologic and pathogenic characteristics of microvascular tissue (41–43). Additionally, oxidative stress, inflammation and endothelial dysfunction are considered the common pathogenic mechanisms of retinal and renal vascular impairment (44, 45). Previously, retinal capillaries have been shown to become sparser as renal function deteriorated. This current study further confirmed that the retinal vasculature became complex and more distorted for those with IRF. Considering that the renal microvasculature is not easily examined *in vivo*, it is reasonable to treat OCTA parameters as a non-invasive tool for diagnosis, treatment planning and clinical surveillance of renal disease.

There are also several limitations. First, the current study was limited by its cross-sectional design, which cannot determine a causal relationship. Longitudinal cohort studies are needed to verify our findings. Second, the absence of other important renal function–related biomarkers, including urinary microalbumin/creatinine ratio and renal pathological biopsy features, limited the deep interpretation of the renal-retinal relationship in the present study. Third, the current study is not a population-based one, and all participants were recruited from certain communities in Guangzhou, and selection bias may exist. However, the results of this article were based on a large homogenous sample after excluding the influence of multiple confounding factors such as eye diseases or invasive treatment history on the results, so we believe that the results of the present study have important reference value. Additionally, renal function, retinal FD and BVT are reported to be vary by region and race (46, 47). Hence, generalization to other areas and ethnicities are limited.

In conclusion, we found that compared to healthy participants, participants with IRF had lower FD and larger BVT, which indicated non-complexity and more tortuously of the retinal microvasculature. Superficial FD was positively associated with eGFR. Our study indicated that OCTA may provide a useful indicator for assessment of renal function and identify those who are at high risk of renal function decline to ensure earlier therapeutic intervention to prevent CKD progression. Further longitudinal studies are needed to confirm the predictive effect of OCTA parameters on renal function decline.

## Data availability statement

The raw data supporting the conclusions of this article will be made available by the corresponding author on reasonable request.

## Ethics statement

The studies involving human participants were reviewed and approved by Ethics Committee of Zhongshan Ophthalmic Center. The patients/participants provided their written informed consent to participate in this study.

## Author contributions

All authors listed have made a substantial, direct, and intellectual contribution to the work, and approved it for publication.
